# Bracket Bonding to All-Ceramic Materials with Universal Adhesives

**DOI:** 10.3390/ma15031245

**Published:** 2022-02-08

**Authors:** Cecilia Goracci, Giuseppe Di Bello, Lorenzo Franchi, Chris Louca, Jelena Juloski, Jovana Juloski, Alessandro Vichi

**Affiliations:** 1 Department of Medical Biotechnologies, University of Siena, 53100 Siena, Italy; giusedibello@gmail.com; 2 Department of Experimental and Clinical Medicine, University of Florence, 50127 Florence, Italy; lorenzo.franchi@unifi.it; 3 Dental Academy, University of Portsmouth, Portsmouth PO1 2QG, UK; chris.louca@port.ac.uk (C.L.); alessandro.vichi@port.ac.uk (A.V.); 4 Clinic for Paediatric and Preventive Dentistry, School of Dental Medicine, University of Belgrade, 11000 Belgrade, Serbia; jelena.juloski@stomf.bg.ac.rs; 5 Department of Orthodontics, School of Dental Medicine, University of Belgrade, 11000 Belgrade, Serbia; jovana.juloski@stomf.bg.ac.rs

**Keywords:** bracket, bonding, universal adhesive, lithium disilicate glass ceramic, monolithic zirconia

## Abstract

The need for bracket bonding to ceramic restorations is increasing. The aim of this study was to evaluate the effect of universal adhesives on bracket adhesion to polished or glazed lithium disilicate (LDS) and monolithic zirconia (MZ) surfaces. One hundred and twenty brackets (N = 10) were bonded to either polished or glazed LDS (e.max CAD B32, Ivoclar Vivadent, Schaan, Liechtenstein) and MZ (In-Ceram^®^ YZ, VITA, Bad Sackingen, Germany) blocks using three different adhesives combined with Transbond™ XT Paste (3M Unitek, Monrovia, CA, USA). Tested universal adhesives were Scotchbond™ Universal Adhesive (SU, 3M St. Paul, MN, USA) and Assure Plus (AP, Reliance, Itasca, IL, USA). Transbond™ XT Primer (XTP, 3M Unitek, Monrovia, CA, USA) served as a control adhesive. Bracket bond strength was measured in shear mode (SBS). Failure type was determined by the Modified Adhesive Remnant Index (ARI). Data were statistically analyzed. On polished LDS, SU yielded bracket SBS significantly superior to those of AP and XTP. On polished MZ, the use of SU and AP significantly enhanced bracket retention as compared with XTP. Low SBS values, below the threshold of clinical acceptability, were reached by all tested adhesives on glazed LDS and MZ specimens. SBS measurements corresponded with failure type observations. Universal adhesives SU and AP could be considered for use on polished LDS and MZ surfaces.

## 1. Introduction

With the constantly growing number of adult patients undergoing orthodontic treatment, the chance for the orthodontist to bond brackets to all-ceramic restorations is also increasing [1,2]. The use of lithium disilicate glass ceramic (LDS) is on the rise, particularly for single-unit crowns [3,4], while for the fabrication of fixed dental prostheses, monolithic zirconia (MZ) has been introduced [5,6,7], both offering a favorable combination of biocompatibility, mechanical and aesthetic properties [3,8,9,10,11]. Using CAD-CAM technologies, all-ceramic restorations are milled from prefabricated blocks finished by either polishing or glazing.

Different surface treatments have been proposed to improve the bond strength of resin composites to ceramic materials [3,12,13]. For LDS ceramic, hydrofluoric acid (HF) etching followed by silanization is considered an efficient method [2,3,14,15,16,17]. An alternative solution to HF etching, such as the application of one-step self-etching ceramic primer, has recently been advocated to promote the adhesion of resin-based materials to LDS, not only to simplify the clinical procedure, but also to eliminate the potential risks of the intraoral use of such a strong acid [3,14,15,18,19,20,21]. While one study reported that this alternative procedure significantly lowered shear bond strengths compared to the standard [14], Vichi et al. showed that self-etching primer (Monobond Etch and Prime, Ivoclar Vivadent, Liechtenstein) had values of immediate bond strength to lithium–silica-based glass ceramics comparable to that of a standard HF etching and silane conditioning protocol [21].

Several mechanical treatments, such as bur roughening [13,22,23], sandblasting with aluminum oxide particles [7,12,22,23,24,25,26,27] and tribochemical silica coating [7,23,24,26], have been proposed for bracket bonding to zirconia. There is, however, the concern that mechanical treatments may alter the surface characteristics of the restoration to such an extent that a complete recovery of the original aesthetics after debonding would not be possible and may also affect the long-term survival of zirconia restorations [23,27,28,29].

It should also be considered that polished and glazed ceramic surfaces provide quite different substrates for bonding, but in the clinical setting, it could be very difficult for the orthodontist to visually distinguish between the two surface finishes.

A recent contribution to the field of adhesive dentistry has been the introduction of so-called ‘universal adhesives’ [30]. The claim for these new materials is that they can function as adhesive primers on several restorative substrates, including LDS and MZ [30,31]. Different results have been reported regarding the success of universal adhesive use for bracket bonding to ceramic materials. The universal adhesive Scotchbond™ Universal (3M ESPE, St. Paul, MN, USA) achieved a higher bracket shear bond strength on the LDS ceramic IPS e.max Press (Ivoclar Vivadent, Liechtenstein) than the control adhesive Transbond™ XT Primer (3M Unitek, Monrovia, CA, USA), applied after substrate silanization, but it was not clear whether the IPS e.max Press ingots surface had originally been polished or glazed [32]. The IPS e.max specimens had been sandblasted with 50 μm aluminum oxide particles prior to the bonding procedure [32], even though sandblasting was shown to reduce the flexural strength of LDS [33,34], and it is not recommended by manufacturers [34]. Furthermore, Scotchbond™ Universal increased the bracket shear bond strength to IPS e.max ZirCAD (Ivoclar Vivadent), a sintered zirconia specimen that was not subsequently polished or glazed [32]. Such an experimental model does not reflect the clinical situation.

Assure Plus (Reliance Orthodontic Products, Itasca, IL, USA) one-step universal primer provided high bracket bond strength to glazed IPS e.max preliminarily subjected to sandblasting with aluminum oxide particles and etched with HF [35], while the bond strength on sandblasted and silanated IPS e.max rods resembled that measured on enamel [36]. The application of universal adhesive Monobond Plus (Ivoclar Vivadent) after tribochemical treatment was considered the most effective conditioning method to enhance the adhesion of orthodontic resin cements to zirconia surfaces in either finishing state [7]. Several other studies did not report whether zirconia specimens had been polished or glazed before the use of Assure Plus [36], Monobond Etch and Prime (Ivoclar Vivadent) [37], and Clearfil Ceramic Primer (Kuraray, Japan) [25] prior to bracket bonding.

Based on these premises, the present study was aimed at assessing whether the use of universal adhesives, in the absence of any mechanical pre-treatment of the substrate, can effectively influence bracket adhesion to polished and glazed lithium disilicate glass–ceramic and monolithic zirconia. The formulated null hypothesis was that two marketed universal adhesives did not significantly differ from each other or from a conventional orthodontic adhesive in their ability to bond metal brackets onto polished or glazed lithium disilicate glass–ceramic and monolithic zirconia CAD–CAM blocks. The bonding ability was assessed with bracket shear bond strength testing and microscopic observations of the debonded surfaces.

## 2. Materials and Methods

### 2.1. Study Protocol

Six blocks of e.max CAD B32 lithium disilicate glass ceramics (Ivoclar Vivadent, Schaan, Liechtenstein, LDS) and six blocks of In-Ceram^®^ YZ T zirconia (VITA Zahnfabrik, Bad Säckingen, Germany, MZ) were utilized for the test. The chemical compositions of the LDS and MZ material are reported in Table 1. Each of the LDS blocks was singularly crystallized according to the manufacturer’s instructions [38], and subsequently, 3 randomly selected blocks were polished, while the other ones were glazed. Each MZ block was cut into two halves before sintering to avoid the risk of fracture under the thermal stress [39]. Six MZ half-blocks chosen at random were polished by the author AV, who operated in strict adherence to the instructions provided by the manufacturer [39], and after calibration, as per the protocol of Antonson et al. [40]. The remaining 6 half-blocks were glazed following the manufacturer’s guidelines [39].

Within each group (N = 10), brackets were bonded horizontally spaced on the same longitudinal block surface of LDS and the same half-block of MZ. After cleansing with ethanol, an oil-free air stream was sprayed over the bonding area for drying. The adhesives Scotchbond™ Universal Adhesive (3M ESPE, St. Paul, MN, USA), Assure Plus (Reliance Orthodontic Products, Itasca, IL, USA), and Transbond™ XT Primer (3M Unitek, Monrovia, CA, USA) were used along with Transbond™ XT Paste (3M Unitek, Monrovia, CA, USA) for bracket bonding. Transbond™ XT Primer, that has been regarded as a standard adhesive for orthodontic purposes, was tested as control material. Table 1 illustrates the chemical composition of the compared adhesives. Experimental groups were defined as reported in Table 2:

One hundred and twenty stainless steel upper incisors brackets (Victory Series 3M Unitek, Monrovia, CA, USA) were chosen at random and distributed among the groups.

Shear Bond Strength

The same experimental protocol as described in a previous paper [41] was followed to calculate the average bracket base, bond the brackets with the different adhesives, subject the bonded specimens to thermocycling, load the brackets to failure and calculate the bracket shear bond strength in MegaPascals (MPa).

ARI examination

**Figure 1:**
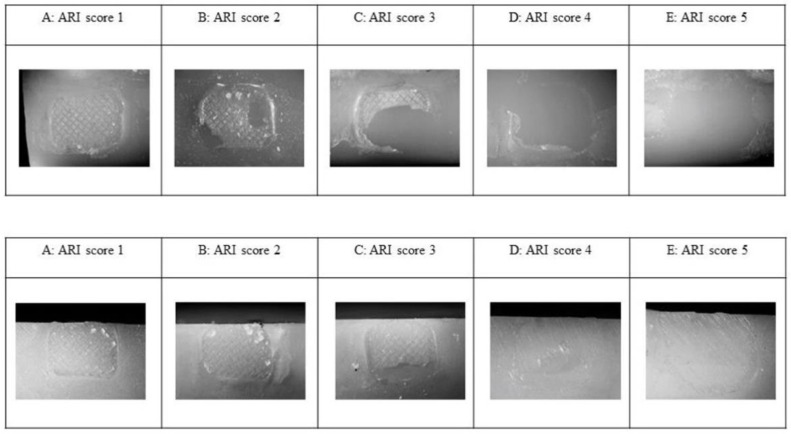
Optical microscope images of lithium disilicate glass ceramics (upper row) and monolithic zirconia (lower row) substrate after bracket debonding (magnification ×20). (**A**–**E**) are representative of 1–5 adhesive remnant index (ARI) scores.

The failure mode was determined by the modified adhesive remnant index (ARI) [42] (Figure 1):

Score (1) all of the adhesive remained on the substrate;

Score (2) more than 90% of the adhesive on the substrate;

Score (3) 10–90% of the adhesive on the substrate;

Score (4) less than 10% of the adhesive on the substrate;

Score (5) no adhesive remained on the substrate.

### 2.2. Statistical Analysis

SigmaPlot for Windows version 11.00 (Systat Software, Inc., San Jose, CA, USA) was used to handle the statistical calculations. In all the analyses, the level of statistical significance was set at *p* < 0.05.

#### 2.2.1. Shear Bond Strength

A preliminary linear regression excluded that the ceramic block per se was an influential factor for the measured bond strengths. Therefore, the bracket provided the statistical unit. Having checked that the conditions of normality of data distribution (Shapiro–Wilk test) and the homogeneity of group variances (Levene test) were met by LDS data, a two-way analysis of variance (ANOVA) was applied with bond strength as the dependent variable, substrate (polished versus glazed) and adhesive type as factors. When indicated, the Tukey test was run for post hoc comparisons.

In the analysis of shear bond strength data from MZ groups, failure to pass the normality test ruled out the use of a two-way analysis of variance (ANOVA). A one-way ANOVA comparing the six MZ experimental groups was therefore tentatively applied. However, the lack of homogeneity of group variances dictated the use of the Kruskal–Wallis test along with Dunn’s Multiple Range test for post hoc analysis.

#### 2.2.2. ARI Score

To assess whether the amount of adhesive remaining on the substrate significantly differed among the experimental groups, two separate Kruskal–Wallis analyses of variance were applied to ARI scores from LDS and MZ groups, and the Dunn’s multiple range test was used for post hoc comparisons.

## 3. Results

### 3.1. Shear Bond Strength

Descriptive statistics of the LDS shear bond strength data are reported in Table 3. The two-way ANOVA revealed that, irrespective of the adhesive, significantly higher bracket bond strengths were achieved on polished than on glazed specimens (*p* < 0.001). Additionally, the adhesive per se was a significant factor for bracket retention (*p* < 0.001). Particularly, the Tukey test disclosed that Scotchbond™ Universal Adhesive yielded significantly higher bond strengths than the other universal adhesive and the control adhesive (*p* < 0.05). Substrate–adhesive interaction was also statistically significant (*p* < 0.001). Specifically, on polished LDS, Scotchbond™ Universal Adhesive provided a significantly stronger bond than the other two bonding systems. The same outcome was observed on glazed LDS. In addition, brackets bonded with Scotchbond™ Universal Adhesive measured significantly higher bond strengths on polished than on glazed LDS surfaces.

Descriptive statistics of MZ shear bond strength data are reported in Table 4. The Kruskal–Wallis test revealed that the experimental groups differed significantly (*p* < 0.05). Particularly, the Dunn’s multiple range test pointed out that on polished MZ, the application of Assure Plus or Scotchbond™ Universal Adhesive significantly enhanced bracket shear bond strength as compared with Transbond™ XT Primer (*p* < 0.05). In contrast, no statistically significant difference in bracket retention was found for glazed MZ, irrespectively of the adhesive (*p* > 0.05). On glazed specimens coated with Assure Plus, the shear bond strengths were significantly inferior to those recorded on polished specimens covered with the same adhesive or with Scotchbond™ Universal Adhesive (*p* < 0.05). In addition, the control adhesive Transbond™ XT Primer obtained significantly stronger adhesion on glazed than on polished MZ (*p* < 0.05).

### 3.2. ARI Scores

Table 5 reports the descriptive statistics of LDS ARI scores. In all the specimens bonded with Transbond™ XT Primer and Assure Plus, adhesive type failures occurred between the adhesive and substrate interface. Conversely, in most of the specimens bonded with Scotchbond™ Universal Adhesive, various amounts of resin composite were retained on the substrate following bracket failure. Subsequently, ARI scores recorded on Scotchbond™ Universal Adhesive specimens were significantly lower than those of the other experimental groups (*p* < 0.05). Substrate pretreatment was not an influential factor for failure type.

Descriptive statistics of MZ ARI scores are presented in Table 6. The Kruskal–Wallis test disclosed the existence of statistically significant differences in ARI score among the experimental groups (*p* < 0.001). In particular, the Dunn’s multiple range test indicated that significantly more resin composite was retained on the substrate when polished specimens were treated with Assure Plus or Scotchbond™ Universal Adhesive (*p* < 0.05). When utilizing Transbond™ XT, all failures were adhesive at the interface between the adhesive and substrate. The same failure type was reported in glazed specimens that had been bonded using Assure Plus.

## 4. Discussion

This study meant to assess whether the use of a universal adhesive as the sole surface pretreatment resulted in clinically satisfactory bracket retention to polished or glazed LDS and MZ substrates. The results led to the rejection of the formulated null hypothesis, as statistically significant differences emerged among tested groups, both in bracket shear bond strength and ARI scores.

This study provided confirmatory evidence to the previous finding that polished and glazed ceramics offer markedly different conditions to bonding [7]. It emerged that Scotchbond™ Universal on polished LDS yielded significantly higher bracket shear bond strengths than those obtained by the other tested universal adhesive (Assure Plus), as well as by the control adhesive (Transbond™ XT Primer). The superior adhesion demonstrated by Scotchbond™ Universal Adhesive may be ascribed to the presence of silane molecules in the bonding agent solution (Table 1). Silane molecules are capable of enhancing adhesion by reacting with exposed hydroxyl groups of LDS on one side and copolymerizing with the bracket bonding material on the other side [14]. The average bond strength provided by Scotchbond™ Universal on polished LDS and by Scotchbond™ Universal and Assure on polished MZ was above the 6–8 MPa interval, which has been indicated as the threshold value for clinically acceptable adhesion in the classic paper by Reynolds [43]. The reference to such threshold values has later been criticized in a systematic review and meta-analysis of in vitro bracket bond strength tests, by remarking that the statement that 6–8 MPa bond strength is adequate for clinical purposes has indeed never been placed under test [44,45]. Moreover, Eliades and Bourauel advised that extrapolating from absolute bond strength values and relating them with an allegedly ‘clinically acceptable’ limit is a questionable procedure [45]. In fact, in vitro bond strengths are measured under specific experimental conditions that invalidate direct between-study comparisons. Only the reference to a previous investigation following the same protocol and using the same testing equipment can be meaningful. In this regard, it should be noted that the bond strengths measured on polished LDS treated with Scotchbond™ Universal Adhesive (9.21 ± 1.80 MPa) were similar to those recorded on enamel by Transbond™ XT Primer/Paste in an earlier investigation using the same experimental setting (9.80 ± 2.28 MPa) [46]. Transbond™ XT Paste has long been used for orthodontic bonding with clinical success [46,47]. The similarity in bracket retention demonstrated by Scotchbond™ Universal Adhesive on polished LDS and by Transbond™ XT Paste on enamel indirectly validates the clinical use of the universal adhesive, enabling the clinician to abandon the use of a potentially toxic conditioner such as HF, eliminating the need to perform a potentially damaging surface mechanical pre-treatment such as sandblasting, and overcoming the necessity to purchase a specific LDS primer. All this reflects a simpler and safer chairside procedure, as well as into a simplification of the inventory assortment.

Bracket bond strengths to LDS of Assure Plus and Transbond™ XT Primer were significantly lower than those achieved by Scotchbond™ Universal regardless of the surface finishing. They both failed to reach adhesion levels that can be considered as compatible with the clinical service [43]. The weakness of the bond established by Assure Plus and Transbond™ XT Primer was also witnessed by the exclusive occurrence of adhesive failures at the substrate site on debonding (Table 4).

On polished MZ, both universal adhesives contributed a significant increase in bracket retention, as compared with the conventional adhesive (Table 3), which could be explained by the 10-MDP monomer contained in the adhesives, acting as an effective adhesion promoter for zirconia [12,24,48,49,50,51]. 10-MDP increases bond strength through chemical bonding between phosphate ester groups of the monomer and hydroxyl groups and metal oxides of zirconia [12,48,49,50]. Even without any mechanical pre-treatment, either universal adhesive attained levels of adhesion that were within the range of clinical acceptability [43]. The opportunity to omit mechanical pre-treatment without compromising bracket retention is clinically advantageous. Additionally, in polished MZ specimens treated with the universal adhesives, the resin composite tended to remain attached to the substrate when the bracket was sheared off (Table 5). This failure pattern, besides confirming that a solid adhesive–substrate bond had been created, was safer for preserving the integrity of zirconia surfaces. The retained resin composite could then be thoroughly removed with proper polishing kits.

Concerning glazed MZ, regardless of the applied adhesive solution, low levels of adhesion, far below the threshold of clinical acceptability [43], were reached. The microscopic observation of the prevalence of failures at the adhesive–substrate interface was in line with the findings of low levels of bracket bond strength.

The present investigation was indeed the first study to distinctly assess the bonding conditions provided by polished and glazed LDS and MZ and to confirm their diversity. Such information had been overlooked by previous research [32]. The clinically relevant finding was that on glazed specimens, regardless of the adhesive and type of ceramic, the measured shear bond strengths were poor and below the value conventionally regarded as the lowest limit for clinical acceptability [43]. According to Kwak et al. [13], whenever it is unclear to the orthodontist whether a glaze layer is on the restoration surface, the latter should be bur ground to eventually remove the glaze and the exposed zirconia substrate should be treated with a zirconia primer prior to bracket bonding. Based on the outcome of the present study, the use of Assure Plus or of Scotchbond™ Universal can be proposed as an alternative to the dedicated zirconia primer for the same purpose. In analogy, lightly grinding the glazed LDS restoration surface and then applying a universal adhesive containing silane molecules, such as Scotchbond Universal™ Adhesive, appears to be a reasonable strategy.

## 5. Conclusions

The use of Scotchbond™ Universal Adhesive enabled performing a clinically reliable bonding procedure on polished LDS and MZ, eliminating the need for any mechanical or chemical pretreatment of the substrate that exposes the ceramic structure to a possibly weakening effect or the patient to a potentially toxic agent, such as fluoridric acid.

## Figures and Tables

**Table 1:** Chemical composition of the tested ceramic blocks and adhesives.

Material	Product Name	Manufacturer	Chemical Composition
Lithium disilicate glass ceramic (LDS)	IPS e.max CAD B32	Ivoclar Vivadent, Schaan, Liechtenstein	In wt%: SiO_2_ 57–80%, Li_2_O 11–19%, K_2_O 0–13%, P_2_O_5_ 0–11%, ZrO_2_ 0–8%, ZnO 0–8%, other and coloring agent 0–12%
Monolithic zirconia (MZ)	In-Ceram^®^ YZ T	VITA Zahnfabrik, Bad Säckingen, Germany	In wt%: ZrO_2_ 90.9–94.5; Y_2_O_3_ 4–6; HfO_2_ 1.5–2.5; Al_2_O_3_ 0–0.3; Fe_2_O_3_ 0–0.3
Universal Adhesive	Scotchbond™	3M ESPE, St. Paul, MN, USA	10-MDP phosphate monomer, Vitrebond copolymer, HEMA, Bis-GMA, dimethacrylate resins filler, silane, initiators, ethanol, water
Universal Adhesive	Assure Plus	Reliance Orthodontic Products, Itasca, IL, USA	Bis-GMA, ethanol, MDP, HEMA
Control Adhesive	Transbond™ XT Primer	3M Unitek, Monrovia, CA, USA	Bis-GMA, TEGDMA, 4-(dimethylamino)-benzeneethanol, camphorquinone, hydroquinone
Light Cure Paste	In-Ceram^®^ YZ T	3M Unitek, Monrovia, CA, USA	Silane treated quartz (70–80% in weight), bisphenol A diglycidyl ether dimethacrylate, bisphenol A bis (2-hydroxyethyl ether) dimethacrylate, silane treated silica, diphenyliodonium

Abbreviations: MDP, methacryloyloxydecyl dihydrogenphosphate; HEMA, 2-hydroxyethyl methacrylate; Bis-GMA, bisphenol A diglycidyl methacrylate; TEGDMA, triethylene glycol dimethacrylate.

**Table 2:** Experimental groups.

Groups	Substrate	Surface Treatment	Adhesive
LDS1	Lithium Disilicate	Polished	Scotchbond™ Universal
LDS2			Assure Plus
LDS3			Transbond™ XT Primer
LDS4		Glazed	Scotchbond™ Universal
LDS5			Assure Plus
LDS6			Transbond™ XT Primer
MZ1	Monolithic Zirconia	Polished	Scotchbond™ Universal
MZ2			Assure Plus
MZ3			Transbond™ XT Primer
MZ4		Glazed	Scotchbond™ Universal
MZ5			Assure Plus
MZ6			Transbond™ XT Primer

**Table 3:** Descriptive statistics of shear bond strength data of lithium disilicate glass ceramics (LDS) groups.

Surface Finishing	Adhesive	N	Mean (MPa)	Standard Deviation (MPa)
**Polished LDS ^A^**	Scotchbond™ Universal	10	9.21 *^aα^*	1.80
	Assure Plus	10	3.62 *^b^*	1.00
	Transbond™ XT Primer	10	4.55 *^b^*	1.07
	Total	30	5.79	2.80
**Glazed LDS ^B^**	Scotchbond™ Universal	10	5.55 ^aβ^	1.80
	Assure Plus	10	3.86 ^b^	0.82
	Transbond™ XT Primer	10	3.67 ^b^	0.85
	Total	30	4.36	1.48
**Adhesive**	**Scotchbond™ Universal ^a^**	20	7.38	2,56
	**Assure Plus ^b^**	20	3.74	0.90
	**Transbond™ XT Primer ^b^**	20	4.11	1.04

Two-way ANOVA, Tukey test (*p* < 0.05). Different superscript letters mark statistically significant differences as follows: different capital bold letters mark the statistically significant difference between glazed and polished substrates per se; different small bold letters mark the statistically significant differences among the adhesives per se; different small letters mark the statistically significant differences among adhesives on glazed LDS; different small italic letters mark the statistically significant differences among adhesives on polished LDS; different Greek letters mark the statistically significant differences between polished and glazed specimens when the brackets were bonded with Scotchbond™ Universal Adhesive.

**Table 4:** Descriptive statistics of shear bond strength data of monolithic zirconia (MZ) groups.

Surface Finishing	Adhesive	N	Mean (MPa)	Standard Deviation (MPa)	Median (MPa)	Interquartile Range (MPa) 25–75%	Significance *p* < 0.05
Polished MZ	Scotchbond™ Universal	10	6.67	0.91	6.66	6.14–7.26	A
	Assure Plus	10	7.34	1.26	7.05	6.64–7.53	A
	Transbond™ XT Primer	10	1.00	0.55	0.86	0.70–1.30	C
Glazed MZ	Scotchbond™ Universal	10	3.99	2.22	3.86	2.18–5.42	ABC
	Assure Plus	10	2.25	0.99	2.02	1.48–2.54	BC
	Transbond™ XT Primer	10	4.41	1.68	4.43	2.92–5.97	AB

Kruskal–Wallis test, Dunn’s multiple range test (*p* < 0.05). In the significance column, different letters label statistically significant between-group differences.

**Table 5:** Descriptive statistics of ARI scores for lithium disilicate glass ceramics groups. In the significance column, different letters label statistically significant differences.

Group	N	Median	Interquartile Range 25–75%	Significance *p* < 0.05
Polished/Scotchbond™ Universal	10	3	2–4	A
Polished/Assure Plus	10	5	5–5	B
Polished/Transbond™ XT Primer	10	5	5–5	B
Glazed/Scotchbond™ Universal	10	4	3–5	A
Glazed/Assure Plus	10	5	5–5	B
Glazed/Transbond™ XT Primer	10	5	5–5	B

Kruskal–Wallis test, Dunn’s multiple range test (*p* < 0.05). In the significance column, different letters label statistically significant differences.

**Table 6:** Descriptive statistics of ARI scores for monolithic zirconia groups.

Group	N	Median	Interquartile Range 25–75%	Significance *p* < 0.05
Polished/Scotchbond™ Universal	10	2	2–3	A
Polished/Assure Plus	10	1	1–2	A
Polished/Transbond™ XT Primer	10	5	5–5	B
Glazed/Scotchbond™ Universal	10	5	5–5	B
Glazed/Assure Plus	10	5	5–5	B
Glazed/Transbond™ XT Primer	10	5	5–5	B

Kruskal–Wallis test, Dunn’s multiple range test (*p* < 0.05). In the significance column, different letters label statistically significant between-group differences.

## Data Availability

The data presented in this study are available on request from the corresponding author. The data are not publicly available due to the university’s policy on access.
